# Supplementary material for: Possible Effects of Early Maternal Separation on the Gut Microbiota of Captive Adult Giant Pandas
Source: Animals (Basel). 2022 Sep 27;12(19):2587. doi: 10.3390/ani12192587 (PMC9559482; doi:10.3390/ani12192587)
Supplement: Supplementary file 1 [file animals-12-02587-s001.zip › Supplement Table.pdf]

Table S1: Species Alpha Diversity Index

SampleID	simpson	chao1	shannon
O_PR_1	0.942619190644	3580.4114	5.778934
O_PR_2	0.982701713232	5024.2211	7.643845
O_PR_3	0.957919053548	4489.5543	6.431899
O_PR_4	0.974593491751	5772.4558	7.524415
O_PR_5	0.974780941474	5649.6124	7.315401
O_PR_6	0.959603204284	3585.5707	6.329429
O_HR_1	0.970625221293	5184.1634	7.219651
O_HR_2	0.964970440085	5186.1696	6.652565
O_HR_3	0.981144309061	5189.6968	7.626753
O_HR_4	0.98043864264	6169.1596	7.626226
O_HR_5	0.97889342516	6530.0134	7.554209
O_HR_6	0.98074416722	6753.0580	7.356411

Table S2. *α* diversity index of gut microbes in captive giant pandas adopting different nursing methods.

**α diversity index**	***p*-value**
Simpson index	0.589
Shannon index	0.589
Chao1 index	0.810
