# Supplementary figures and images for: Possible Effects of Early Maternal Separation on the Gut Microbiota of Captive Adult Giant Pandas
Source: Animals (Basel). 2022 Sep 27;12(19):2587. doi: 10.3390/ani12192587 (PMC9559482; doi:10.3390/ani12192587)

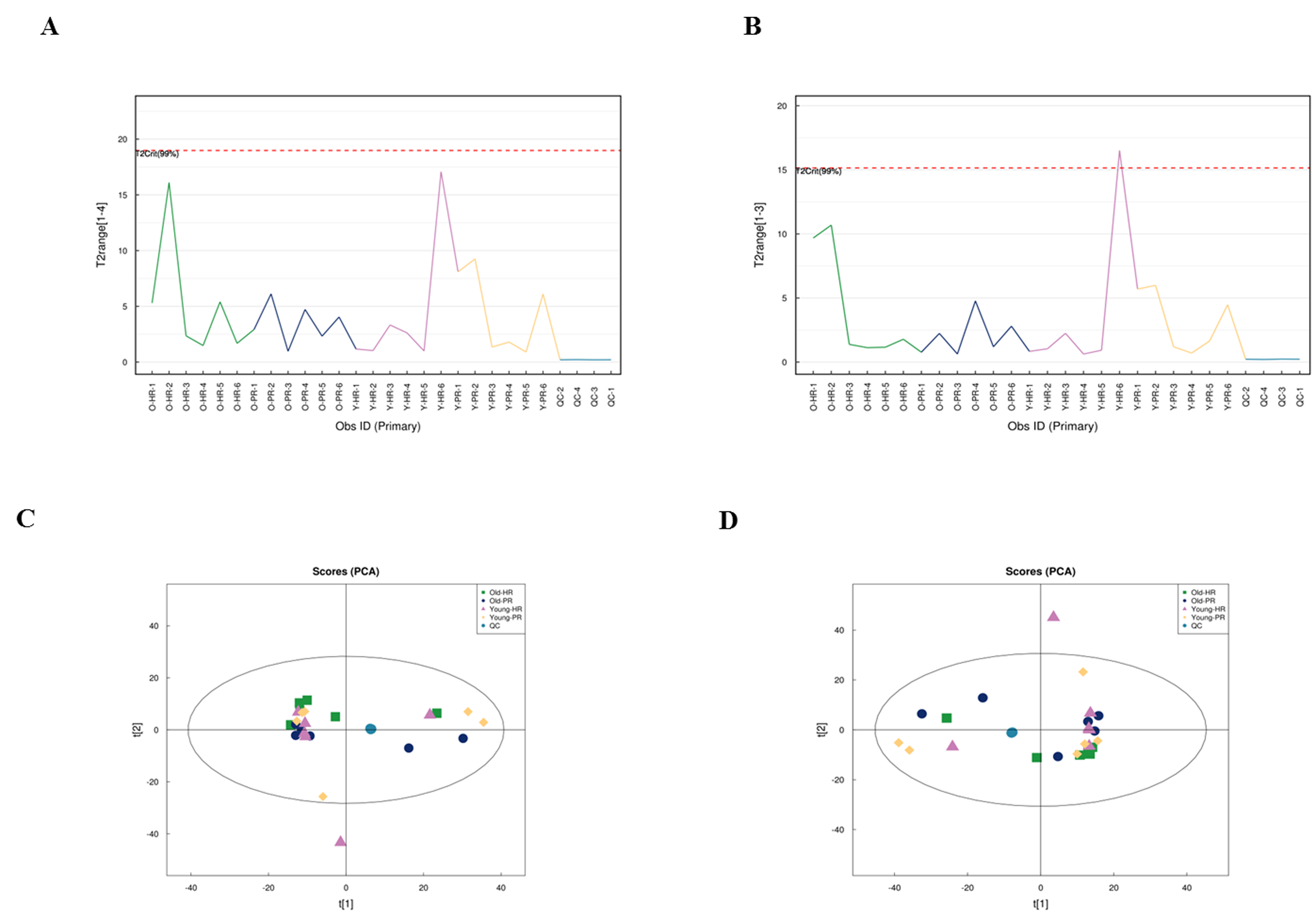

Supplement: Supplementary file 1 [file animals-12-02587-s001.zip › Figure S1.tif]

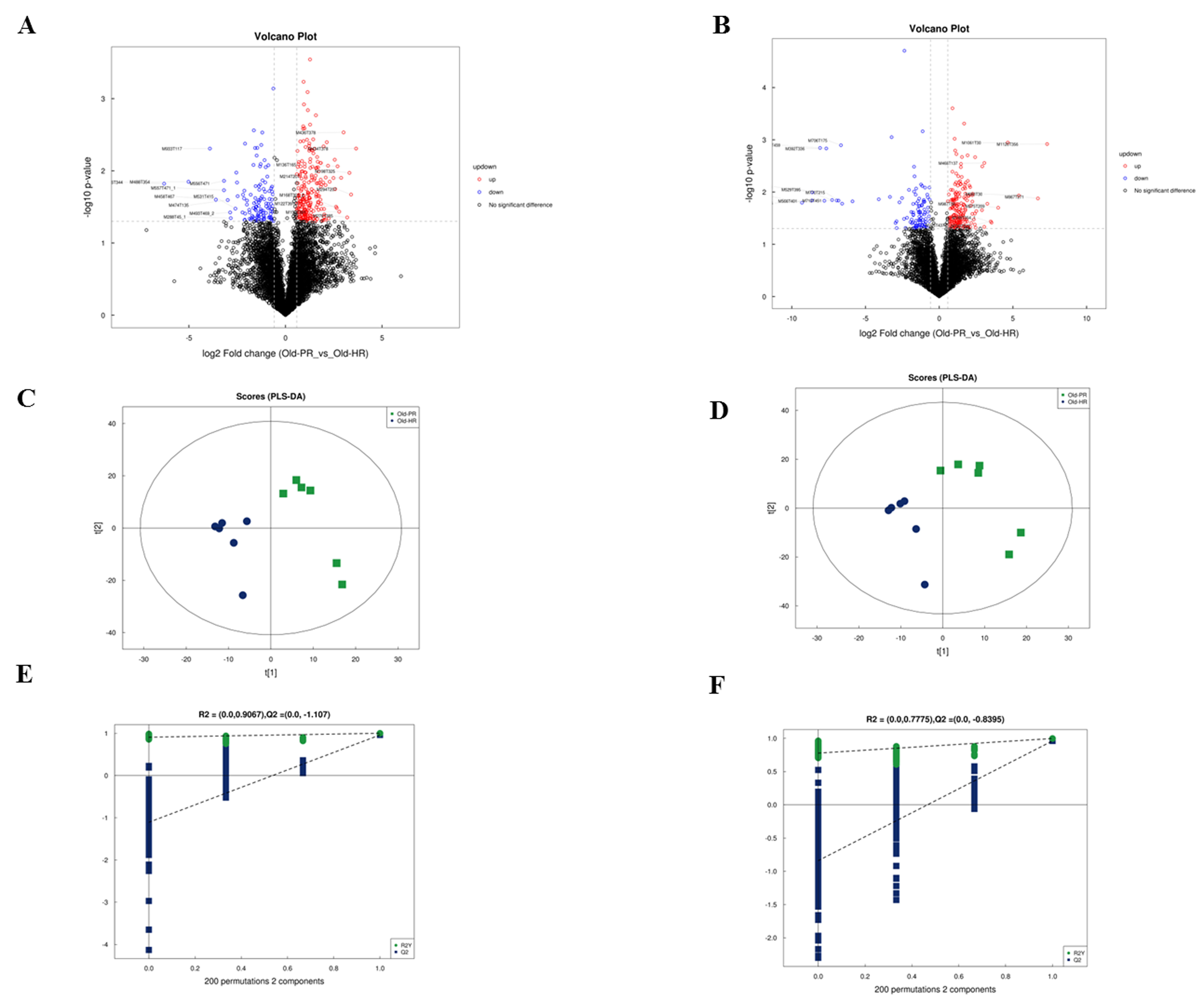

Supplement: Supplementary file 1 [file animals-12-02587-s001.zip › Figure S2.tif]
